# The Anticancer Properties of *Herba* *Epimedii* and Its Main Bioactive Componentsicariin and Icariside II

**DOI:** 10.3390/nu8090563

**Published:** 2016-09-13

**Authors:** Meixia Chen, Jinfeng Wu, Qingli Luo, Shuming Mo, Yubao Lyu, Ying Wei, Jingcheng Dong

**Affiliations:** 1 Department of Integrative Medicine, Huashan Hospital, Fudan University, Shanghai 200040, China; cmx023@126.com (M.C.); qingqingluo2010@163.com (Q.L.); moshuming0703@163.com (S.M.); lvyubao80313@163.com (Y.L.); weiying_acup@126.com (Y.W.); 2 Department of Dermatology, Huashan Hospital, Fudan University, Shanghai 200040, China; wujinfeng21@163.com

**Keywords:** anticancer properties, *Herba Epimedii*, icariin, icariside II

## Abstract

Cancer is one of the leading causes of deaths worldwide. Compounds derived from traditional Chinese medicines have been an important source of anticancer drugs and adjuvant agents to potentiate the efficacy of chemotherapeutic drugs and improve the side effects of chemotherapy. *Herba* *Epimedii* is one of most popular herbs used in China traditionally for the treatment of multiple diseases, including osteoporosis, sexual dysfunction, hypertension and common inflammatory diseases. Studies show *Herba* *Epimedii* also possesses anticancer activity. Flavonol glycosides icariin and icariside II are the main bioactive components of *Herba* *Epimedii*. They have been found to possess anticancer activities against various human cancer cell lines in vitro and mouse tumor models in vivo via their effects on multiple biological pathways, including cell cycle regulation, apoptosis, angiogenesis, and metastasis, and a variety of signaling pathways including JAK2-STAT3, MAPK-ERK, and PI3k-Akt-mTOR. The review is aimed to provide an overview of the current research results supporting their therapeutic effects and to highlight the molecular targets and action mechanisms.

## 1. Introduction

Cancer is a complex genetic disease involving abnormal cell growth and is continue to be one of the major causes of deaths in both developed and developing countries [1]. According to statistics, there are approximately 14.1 million new cancer cases and 8.2 million deaths in 2012 [2]. It is estimated that new cancer cases will increase to 20 million by 2025. Cancer has been recognized as one of the most crucial health problems all over the world due to its great increased incidence and significant mortality.

Plants have a long history of use in the treatment of cancer and it is reported that more than 3000 plant species have been used [3,4]. The search for anticancer drugs from plant sources started as early as the 1950s, and at present over 60% of anticancer drugs currently used are derived directly or indirectly from natural sources, including plants, marine organisms and micro-organisms [5,6]. One of the successful stories is the discovery and development of the vinca alkaloids, vinblastine and vincristine, isolated from *Catharanthusroseus G. Don* (Apocynaceae) [7], which are the first plant-derived anticancer agents applied to clinical use for the treatment of various cancers [8,9].

**Figure 1:**
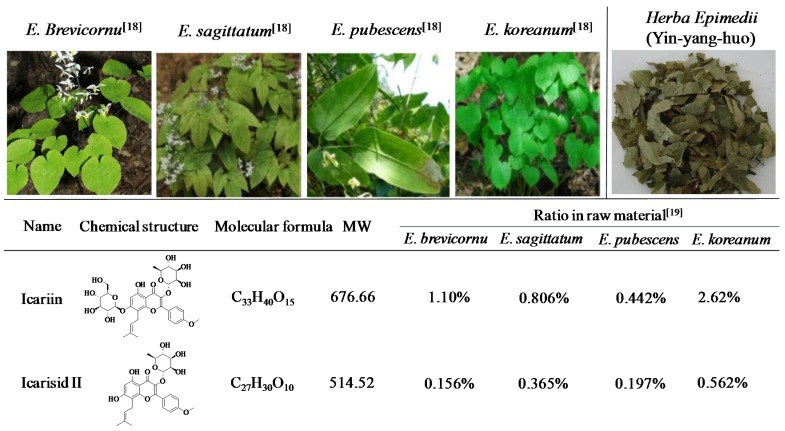
Natural sources and chemical structures of icariin and icariside II [18,19]. Herba Epimedii is made up of the dried leaves of *E.* *brevicornu*, *E.* *sagittatum*, *E.* *pubescens* or *E.* *koreanum*.

*Herba* *Epimedii* (Common name: Yin-yang-huo in China, Figure 1) is the dried leaf of *Epimedium* *brevicornu* Maxim., *Epimedium* *sagittatum* (Sieb. EtZucc.) Maxim., *Epimedium* *pubescens* Maxim. or *Epimedium* *koreanum* Nakaias as recorded in the Chinese Pharmacopoeia (Figure 1) [10]. There are more than 40 kinds of *Epimedium* plants all over the world, mainly distributed in the southwest and central regions of China, although some are found in the temperate and subtropical regions of Asia, Middle East as well as Europe [11]. *Herba* *Epimedii* has been recorded in the Chinese medical classics *Shen* *Nong Ben Cao Jing* 400 years ago and has been used in various traditional Chinese formulations. The herb is believed to “nourishing the kidney and reinforcing the Yang” and is proven to have remarkably therapeutic activities. In China and Japan, *Herba* *Epimedii* alone, or in the formulations, have been widely used for treatment of osteoporosis [12,13], sexual dysfunction [14], hypertension [15] and common inflammatory diseases, such as chronic obstructive pulmonary disease [16]. In addition, *Herba* *Epimedii* has been shown to exert anticancer effect on cancer cell lines in vitro and also in vivo in mouse tumor model [17].

More than 260 constituents have been detected from Herba Epimedii with 141 flavonoids, 31 lignins and multiple other kinds of compounds [11], and the flavonoid glycosides have been confirmed to be the fundamental pharmacologically active constituents [20]. Icariin (Figure 1) is the major active constituent and has been chosen as the chemical marker for quality control of *Herba* *Epimedii* in Chinese Pharmacopeia. It is specified that the contents of icariin and the total flavonoids are no less than 0.5% and 5.0%, respectively [10]. Icariin has been found to possess a variety of pharmacological activities, including anti-osteoporosis [21], anti-inflammatory [22,23], antioxidant [24], antihepatotoxic [25], antidepressant [26] and neuroprotective effects [27]. It was also demonstrated to improve sexual disorder and to protect against cardiac ischemia/reperfusion injury [28,29] and atherosclerosis [30]. Besides, icariin was reported to exhibit anticancer activity against a series of human cancer cell lines.

Icariside II (Figure 1), another active flavonoid glycoside derived from *Herba* *Epimedii*, is the major pharmacological metabolite of icariin in vivo and has been reported to be obtained from icariin through enzymatic hydrolysis [31]. Icariside II has been shown to promote osteogenic differentiation of bone marrow derived stromal cells [32]. It shows protective effects on cognitive deficits [33] and cerebral ischemia-reperfusion injury [34] at least in part due to the inhibition of NF-kappaB. Additionally, icariside II has also been demonstrated to possess cytotoxic and cytostatic activities against various cancer cell lines.

The topic of this review will emphasize on the antitumor activities of *Herba* *Epimudii* and its two main active constituents and the mechanisms of action discovered so far.

## 2. Anticancer Effects of *Herba* *Epimedii* in Vitro and in Vivo

Recent studies showed that *Herba* *Epimedii* could restrain the proliferation of human breast cancer cell lines in vitro as well as inhibit tumor growth in rat model of bone metastasis from breast cancer. The antiproliferative activities of the ethanol extracts from *Herba* *Epimedii* on two different types of human breast cancer cells were investigated [35]. The 95% ethanol extract significantly inhibited the proliferation of human breast cancer MCF-7 cells in the range of 100–800 μg/mL in a dose dependent manner with an IC_50_ of 528 μg/mL after 72 h treatment. The 70% ethanol extract exhibited a certain activity on the growth of MCF-7 cells, whereas the 20% and 40% ethanol extracts showed no significant antiproliferation activity. The four different ethanol extracts of *Herba* *Epimedii* showed no obvious antiproliferative activity on human breast cancer MDA-MB-231 cells. In the rat model of bone metastasis from breast cancer, significant increase of 50% Paw withdrawn threshold (50% PWT) and reduction of tumor sizes were observed after oral administration of the decoction of *Herba* *Epimedii* at a dose of 5 g/kg daily for 20 days. In addition, the bone structural mineral density (BMD) and bone mineral capacity (BMC) were significantly enhanced [36]. In another study, it was found that the *Epimedium* *sagittatum* extract inhibited the proliferation of various hepatoma and leukemia cell lines, including SK-Hep1, PLC/PRF/5, K562, U937, P3H1 and Raji, with IC_50_ values of 15, 57, 74, 221, 40 and 80 μg/mL, respectively, whereas it showed no inhibition effects to HepG2 and Hep3B cell lines (IC_50_ > 500 μg/mL). The Hep3B was found to be less sensitive to the extract compared with other cell lines, consistent with the reported result that cells with the p53 gene deleted, just like Hep3B cell line, were more resistant to drugs [17].

## 3. Anticancer Effects of Icariin and Icariside II in Vitro and in Vivo

As for anticancer effects, studies have been performed in various human cancer cell lines. Icariin and icariside are proved to inhibit the growth of human cancer cells in vitro through intervening with multiple signaling pathways which are crucial to tumor growth, progression, invasion and apoptosis. Different concentrations of icariin and icariside II were used in the studies, depending on the types of cancer cell lines. The anticancer activity of icariin and icariside II and the molecular targets on various cancer cell lines were summarized in Table 1.

Icariin and icariside II have been demonstrated to exhibit in vivo suppressive effects both on tumor weight and tumor volume on a variety of mouse tumor models without obvious side effects (Table 2). Intraperitoneal administration of icariin at the doses of 15–150 mg/kg significantly inhibited tumor growth in xenografted mice models with almost no or very low toxicity to animals. Oral administration of 65 mg/kg icariin was shown to inhibit the growth of melanoma tumor and to inhibit the metastasis of B16 melanoma cells, and the lifespan was apparently pro-longed [45]. At the doses of 10–100 mg/kg, icariside II resulted in significant decrease in tumor weight and volume in cancer cell bearing mice models. All these data suggested that icariin and icariside II indeed have therapeutic potential against cancers.

## 4. Icariin and Icariside II as Adjuvant Therapy

While icariin and icariside II as single agent exhibited antitumor activities towards diverse human cancers, their potentials of potentiating the antitumor activity of a variety of chemotherapeutic drugs as adjuvant agents have shown perspectives in recent years. The combination treatment with the natural bioactive components and the chemotherapeutic drugs could facilitate chemotherapy for patients with cancers and provide a higher efficacy remedy.

Icariin has been shown to potentiate the antitumor activity of arsenic trioxide, temozolomide, doxorubicin, 5-fluorouracil and gemcitabine on a variety of human cancer cell lines, including acute promyelocytic leukemia, glioblastomamultiforme, hepatocellular carcinoma, osteosarcoma, colorectal cancer and gallbladder cancer cell lines, as well as in xenograft murine models (Table 3).

Arsenic trioxide (ATO), a traditional Chinese medicine, has been wildly used for the treatment of acute myeloid leukemia (AML) since 1970s in China and has been recommended as the front-line agent. In addition, ATO has exhibited antitumor activity against various solid tumor cell lines. Icariin has demonstrated to potentiate the antitumor activity of ATO in treating AML and hepatocellular carcinoma, at least partially correlated with the increase in the accumulation of intracellular reactive oxygen species (ROS). Of note, the sensitivity of APL cell line NB4 to ATO dues to the reduced glutathione content and the increased ROS production. Adjuvant agent, which promotes the accumulation of ROS, such as icariin, could potentiate the antitumor activity of ATO against APL [63]. Moreover, co-treatment with ATO and icariin resulted in a significant inhibition of tumor growth in xenograft murine model of Hep G2 compared to the treatment with either agent alone by promoting the generation of ROS and suppressing NF-κB without systemic toxicity [64].

Icariin potentiated the anti-tumor activity of temozolimide in glioblastomamultiforme cell line U87MG [62]. The cytotoxicity of doxorubin was enhanced by icariin via the inhibition of ABC1 and down regulation of PI3K/Akt pathway [65]. The combination of icariin and 5-fluorouracil led to the inhibition of tumor growth by suppressing NF-κB activity [66]. Icariin also potentiated the anti-tumor activity of gemcitabine in the treatment of gallbladder cancer by inhibiting NF-κB [67].

Icariside II has been demonstrated an increased inhibitory effect on human melanoma cells and multiple myeloma U266 cells when combined with paclitaxel and bortezomib/thalidomide, respectively (Table 3).

Paclitaxel is a widely used cancer chemotherapeutic drug and exhibits antitumor activity in a variety of human malignancies. It was revealed to induce the activation of the toll-like receptor 4 (TLR4) signaling pathway, which was functionally associated with tumor growth, invasion and chemoresistance [68]. Inhibition of the activation of TLR4-MyD88 has been considered as a novel approach for reversing chemoresistance of paclitaxel. Data from our laboratory demonstrated that Icariside II enhanced paclitaxel-induced apoptosis in human melanoma cells, which might be due to its inhibition on paclitaxel-induced activation of TLR4-MyD88-ERK signaling [48].

Bortezomib (a proteasome inhibitor) and thalidomide (an inhibitor of TNF expression) have been reported to be used for the treatment of multiple myeloma patients. Icariside II has been found to induce the suppression of survival proteins such as Bcl-xL and surviving as well as cleavages of PARP and caspase-3. Co-treatment of icariside II with bortezomib or thalidomide significantly improved the cytotoxic effects of bortezomib and thalidomide from 25% and 20% to 60% and 50% in U266 cells, respectively, accompanied by the further increase of caspase-3 activation and PARP cleavage [59].

Icariin and icariside II have played an excellent adjuvant effects without causing systemic toxicity in the chemotherapeutic treatment of several tumors by interfering with multiple molecular targets in tumor cells. They might be considered as the potential candidates for treating tumors in combination with common chemotherapeutic drugs, thus contributing to the development of the successful therapeutic strategies.

## 5. Molecular Mechanisms of Anticancer Activity of Icariin and Icariside II

**Figure 2:**
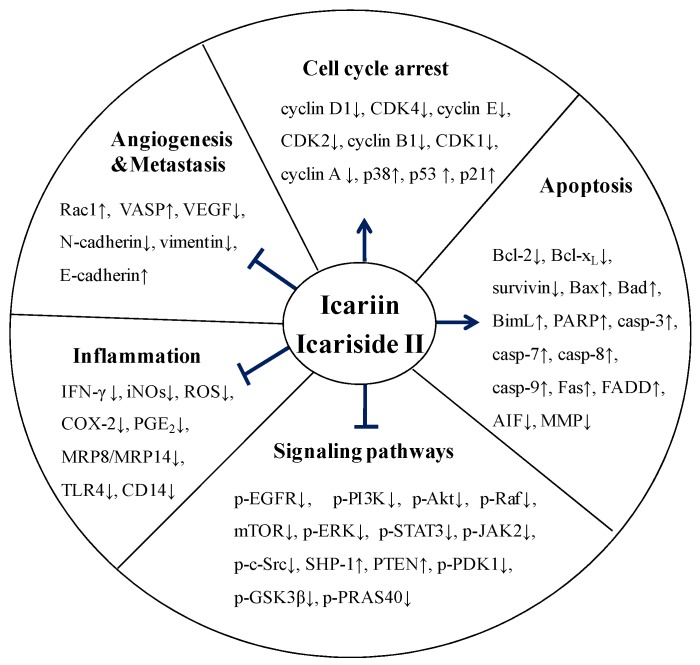
Overview of the anti-cancer effects of icariin and icariside II. Icariin and icariside II stimulate the cell cycle arrest via upregulation of p38, p53, and p21. Icariin and icariside II are involved in the induction of apoptosis and inhibit tumor angiogenesis and metastasis via suppression of multiple signaling pathways. They also have anti-inflammatory effects via downregulation of several factors, such as IFN-γ, iNOs, and COX-2, (← activation; ⊥ inhibition; ↑, up-regulation; ↓, down-regulation).

Multiple studies have been performed to investigate the mechanisms by which icariin and icariside II exert their anti-tumor effects. The mechanisms are comprehensive and diverse, involving the regulation of a variety of targets, which play a particularly important role in the process of tumor proliferation, invasion, angiogenesis, metastasis and apoptosis. Besides the effects of icariin and icariside II on numerous signaling proteins and transcription factors, they also regulate the stages of the tumor cells by inhibiting proliferation and inducing apoptosis. In addition, they could play their anti-tumor effect through improving the tumor inflammatory microenvironment (Figure 2). Icariin and icariside II might be considered as potential chemotherapeutic agents for the treatment of various human cancers due to their multiple targets on the cell growth processes.

### 5.1. Effect on Cell Cycle Regulation

Cell division is divided into two stages: Mitosis (M) and the interlude between two M phases, including G1, S, and G2 phases. In addition, cells in G1 can enter a resting state called G0 just before the DNA replication [69,70]. In normal cells, the cell cycle is a highly regulated event, which is accurately regulated by cyclin dependent kinases (CDKs). The CDKs interact with various corresponding cyclins to form active complexes to ensure that the process at each stage is completely accomplished [71]. Tumor suppressor gene p53 is a vital cell cycle checkpoint regulator at the G1/S and G2/M checkpoints and serves to monitor the accuracy of vital events [72,73]. p38 MARK activation leads to the accumulation of p53 and the p53-induced cell cycle arrest is primarily mediated by the activation of p21 [74].

**Figure 3:**
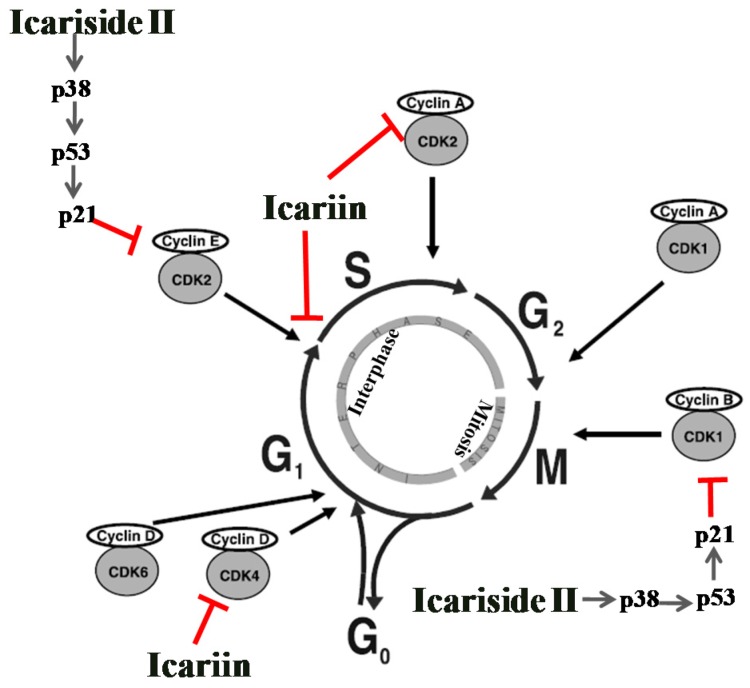
The cell cycle arrest induced by icariin and icariside II. Icariin and icariside II stimulate cell cycle arrest via suppression of the CDKs and cyclins at different stages, (← activation; ⊥ inhibition).

As shown in Figure 3, there are five active CDKs during the cell cycle, CDK2, CDK4 and CDK6 for G1, CDK2 for S, and CDK1 for both G2 and M. The complexes of cyclin D with CDK4 or CDK6 promote the progression through G1, cyclin E/CDK2 activates G1 into S, cyclin A/CDK2 promotes S progression, cyclin A/CDK1 activates G2 into M, while cyclin B/CDK1 stimulates the mitotic phase [75]. Disorders in the regulatory control of cell cycle could lead to the formation of tumors, characterized by the unlimited cell proliferation and abnormal apoptosis. Overexpression of cyclin D1 has been recognized as one of the distinct features in many types of human tumors [76]. Suppression of deregulated cell cycle progression in cancer cells by inhibiting the activities of CDKs or the cyclins have been considered as an effective strategy to inhibit the proliferation of the tumors [77].

Icariin has been reported to arrest the cell cycle at G0/G1 phase on the HepG2 cells. Icariin treatment for 72 h significantly increased the proportion of cells in G0/G1 phase, while the proportion of cells in S phases was remarkably lower (21.07%, *p* < 0.01) than that of the control group (28.62%) (Figure 3) [37]. Icariin also showed a weak G1 phase arrest accompanied by a mitochondrial transmembrane potential drop by decreasing cyclin D1 and CDK4 in human prostate carcinoma PC-3 cell line [39]. In another report, icariin stimulated S phase arrest in medulloblastoma cells by regulating the cell cycle regulators cyclin A, CDK2 and cyclin B1 [51].

Our study demonstrated that icariside II induced cell cycle arrest at G0/G1 and G2/M phases in A375 human melanoma cell line (Figure 3). After treatment with icariside II at a series of concentrations 0, 25, 50 and 100 μM, the percentage of cells in G0/G1 phase significantly increased with the increase of icariside II concentration and peaked at 100 μM (69.51%), as compared to the control group (44.01%). The cell cycle arrest of A375 cells at G0/G1 phase was found to be correlated with the decreased expression of cyclin E and CDK2, and the arrest at G2/M phase was associated with decreased expression of cyclin B1/CDK1 complex. It was determined that IS inhibits cell proliferation and induces cell cycle arrest through the generation of reactive oxygen species and activation of p38 and p53. These findings were further supported by the evidence that pretreatment with N-acetyl-L-cysteine, SB203580 or pifithrin-α significantly blocked icariside II-induced reduction of cell viability, increase of cell death and cell cycle arrest. Crucially, it was confirmed that these effects were mediated at least in part by activating the ROS-p38-p53 pathway [46].

### 5.2. Effect on Apoptosis

Apoptosis occurs normally during development to maintain the cell population in normal tissues or occurs as a defense mechanism to selectively eliminate the defective or unwanted cells which are damaged by disease [78]. It has been recognized as a necessary complementary to proliferation and plays a vital role in the development and regulation of the immune system, as well as the removal of damaged cells, and what’s more, the disruption of apoptosis is implicated in tumors development [79].

**Figure 4:**
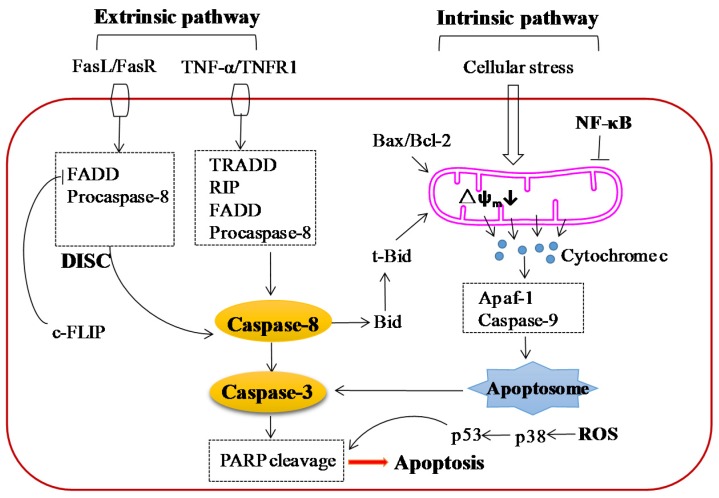
Apoptosis signaling pathways induced by icariin and icariside II. Icariin and icariside II induce apoptosis of tumor cells through two pathways, extrinsic (receptor-mediated) pathway and intrinsic (mitochondria-mediated) pathway. The binding of TNF-α with TNFR1 leads to the formation of TRADD and RIP, then the two factors combine with FADD and procaspase-8, which are resulted from FasL/FasR pathway, leading to the formation of death inducing signaling complex (DISC) and activating caspase-8. Activated caspase-8 stimulates the downstream caspase-3 and PARP, resulting in apoptosis, or cleaves Bid, which is the link between extrinsic pathway and intrinsic pathway, leading to the activation of intrinsic pathway. The cellular stress, activation of t-Bid, or modulation of Bax/Bcl-2 result in the downregulation of mitochondrial membrane potential and subsequent release of cytochrome c. Once released to cytosol, cytochrome c interacts with Apaf-1, resulting in the activation of caspase-9, which then activates caspase-3 resulting in cell death. Activation of NF-κB leads to the activation of several anti-apoptotic factors, which subsequently block the mitochondria-mediated pathway. Tumor cells produce ROS at higher levels, leading to the activation of p38 and p53, and subsequent apoptosis, (← activation; ⊥ inhibition).

It is well established that there are two main apoptotic pathways: The extrinsic (receptor mediated) and the intrinsic (mitochondrial mediated) signaling pathway (Figure 4). The extrinsic signaling pathway is initiated by the ligation of ligands and corresponding death receptors of the tumor necrosis factor (TNF) receptor subfamily, such as FasL/FasR and TNF-α/TNFR1 [80]. The formation of death-inducing signaling complex (DISC) leads to the autocatalytic activation of procaspase-8 [81]. The intrinsic apoptotic pathway is triggered by cellular stress factors including the negative signals involving the absence of some growth factors, hormones and cytokines and positive signals, including radiation, toxins, hypoxia, viral infections, and free radicals. These stimuli at on the mitochondrial membrane and result in change of themitochondrial permeability transition (MPT), loss of the mitochondrialtransmembrane potential and release of the pro-apopto ticproteins such as cytochrome c and apoptosis inducing factor (AIF) from mitochondria into the cytosol [82]. The released cytochrome c activates Apaf-1 and procaspase-9, resulting in the formation of apopto some [83]. The mitochondrial mediated apoptotic pathway is regulated by the members of the Bcl-2 family proteins [84], which are reported to be implicated with the tumor suppressor protein p53 and comprise two groups of proteins with the opposite function, the anti-apoptotic proteins (e.g., Bcl-2, Bcl-x and Bcl-x_L_) and the pro-apoptotic proteins (e.g., Bax, Bid, Bak, Bim and Bik) [85]. The extrinsic pathway can be linked with the intrinsic pathway through caspase-8 by inducing the activation of Bid, in turn, acting on the mitochondrial membrane leading to the release of cytochrome c (Figure 4) [86].

Multiple studies have shown that icariin and icariside II can selectively kill cancer cells without apparent toxicity on normal cells by inducing apoptosis in many cancers, including hepatocellular carcinoma, prostate carcinoma, esophageal squamous cell carcinoma, ovarian cancer, lung cancer, melanoma, leydig cell tumor, gastric adenocarcinoma, promyelocytic leukemia, sarcoma, hepatoblastoma, medulloblastoma, osteosarcoma, epidermoid carcinoma, acute myeloid leukemia, breast cancer, and multiple myeloma. It seems that icariin and icariside II induce apoptosis via both extrinsic (caspase induced) and intrinsic (mitochondrial induced) signaling pathways.

Icariside II has been reported to increase the expression of Fas and the Fas-associated death domain (FADD) without changing the level of Daxx, which is also an Fas binding protein inducing apoptosis by activating the JNK pathway, and activate caspase-8 and caspase-3 in MCF-7 breast cancer cells, indicating the involvement of extrinsic apoptosis pathway [58]. It was speculated that the apoptosis induced by icariside II might be occurred by stimulating Fas/FADD signaling independently of FasL and subsequently activating caspase-8. Moreover, the extrinsic signaling pathway might exert its effects on the icariside II-treated MCF-7 cells independent of the intrinsic pathway for the absence truncated Bid (tBid), which is the key molecule linking the two apoptosis pathways.

Several studies have revealed that icariin and icariside II exert their anti-tumor effects by inducing apoptosis via mitochondrial induced signaling pathway, mainly characterized by the loss of mithchondrial membrane potential, release of cytochrome c, activation of caspase-9, caspase-3 and PARP, and elevation of intracellular reactive oxygen species (ROS), which played vital role in cell apoptosis (Table 1). In addition, icariside II has also been shown to induce the release of the apoptosis-inducing factor (AIF) and cytochrome c, and the activation of caspase-9, which are characteristic features of intrinsic apoptosis pathway [58].

Nuclear factor-kappaB (NF-κB), an inducible transcription factor, and its downstream genes were suggested to play an important role in cell proliferation, invasion, apoptosis and chemoresistance [87]. Recent studies have shown that icariin exhibits antitumor activity and potentiates the antitumor effect of several chemotherapeutic drugs via down-regulation of NF-κB in various human cancer cells, generally accompanied by the down-regulation of Bcl-2 and Bcl-x_L_ [62,64,66,67]. Icariside II has been shown to prohibit invasion of lung cancer A549 and H1299 cells through suppression of Akt/NF-κB pathway [44].

### 5.3. Effect on Angiogenesis and Metastasis

The formation of new blood vessel is one of the key factors responsible for tumor growth and metastasis, not only supply nutrients for the metabolic needs of rapidly proliferating cancer cells but also provide conditions for cancer spread, resulting in malignant tumor growth, invasion and metastasis. Microvascular density in primary tumors is implicated in the numbers of tumor cells entered into the circulation in many tumors. Angiogenesis is regulated by multiple pro-angiogenic genes and signaling molecules including vascular endothelial growth factor (VEGF), basic fibroblast growth factor (bFGF), epidermal growth factor (EGF), platelet-derived growth factors (PDGF), angiopoetin (Ang), hypoxia-inducible factors, and matrix metal oproteinases [88].

Icariin has been shown to significantly prohibit the proliferation of vascular smooth muscle cells (VSMCs) and the activation of ERK1/2. Moreover, icariin also induced G1/S phase cell cycle arrest and decreased the expression of PCNA in VSMCs [89]. Based on these results, it was proposed that the inhibitory effect of icariin on the proliferation of VSMCs might be responsible for the suppression of tumor metastasis.

Effects of icariin and icariside II treatments on the adhesion and metastasis have been investigated in various human tumor cells, including esophageal carcinoma EC109 and TE1 [41], gastric adenocarcinoma BGC-823 [50], and lung cancer A549 and H1299 cell lines [44].

The adhesion and migration of icariin-treated EC109 and TE1 cells were evaluated after incubation in 15 μM ICA for 24 h [41]. The cell adhesion ratio decreased significantly to 47.23% ± 8.97% of that of the control in EC109 cells and 45.98% ± 6.72% of that of the control in TE1 cells, respectively (*p* < 0.05). Similarly, the scratch wound distance significantly increased by 159.23% ± 13.27% in EC109 cells and 179.26% ± 15.14% in TE1 cells, respectively (*p* < 0.05) [41]. Icariin was also reported to significantly inhibit tumor cells migration and invasion of human gastric adenocarcinoma cell line BGC-823 via the down-regulation of Rac1 and VASP [50]. The combination of icariin and the selective siRNA targeting Rac1 and VASP promoted the inhibitory effects. In addition, transfection with Rac1 plasmids pcDNA3-EGFP-Rac1-Q61L resulted in the improvement of expression levels of both Rac1 and VASP. Based on these results, it could be concluded that icariin inhibit the tumor cell invasion and migration through the Rac1-dependent VASP pathway [50]. A study has showed that icariside II prohibited the migration and epithelial-mesenchymal transition (EMT) via the inhibition of N-cadherin and vimentin up-regulation, and E-cadherin down-regulation induced by THP-1-CM in lung cancer A549 and H1299 cells [44].

### 5.4. Effect on Multiple Signaling Pathways

Multiple signaling pathways played vital role in cell survival, apoptosis and metastasis. Targeting various signaling pathways has been considered as a successful option in the treatment of cancer for its potential to avoid drug resistance, which is one of the major drawbacks of most anticancer drugs. Epidermal growth factor receptor (EGFR) is overexpressed in various types of human cancers and its expression is implicated with poor clinical prognosis [90]. Several small molecular kinase inhibitors and antibodies targeting on EGFR have been approved by FDA to treat diverse types of tumors. Activated EGFR recruits a variety of downstream signaling molecules, leading to the activation of major signaling pathways such as JAK2-STAT3, MAPK-ERK, and PI3k-Akt-mTOR, which play vital role in tumor growth, progression, and survival [91].

The inhibitory effects of icariside II on EGFR signaling have been investigated in human epidermoid carcinoma A431 cells [56] and in human osteosarcoma MG-63 and Saos-2 cells [54]. In our study, icariside II was found to inhibit the cell viability of A431 cells, accompanied by the decrease of phosphorylated EGFR. Pretreatment with LY294002 (a phosphatidylinositol 3-kinase (PI3K) inhibitor), EGF (an EGFR agonist) and AG1478 (an EGFR inhibitor) partially reversed the icariside II‑induced decrease in cell viability, indicating icariside II effectively inhibited the EGF-induced activation of the EGFR pathway [56]. Icariside II was also found to decrease cell proliferation in MG-63 and Saos-2 cells by inactivating EGFR/mTOR signaling pathway and the decreased cell viability could be reversed partially by the pretreatment of EGF [54].

STAT3 is activated in a broad spectrum of human cancers, such as prostate cancers [92], breast cancer [93], and nasopharyngeal carcinoma [94], and has been implicated as a potential therapeutic target for multiple human cancers. Icariside II exhibited inhibitory effect on STAT3 signaling pathway in human melanoma A375 and SK-MEL-5 cells [47], epidermoid carcinoma A431 cells [56], acute myeloid leukemia U937 cells [57] and multiple myeloma U266 cells [59].

The phosphorylation of STATs is principally mediated via the activation of non-receptor protein tyrosine kinases called as JAK. Data from our laboratory showed that Icariside II dramatically inhibited the proliferation of melanoma A375 and SK-MEL-5 cells in vivo and in vitro by inhibiting the activation of the JAK-STAT3 and MAPK pathways but promoting an unsustained activation peak of the PI3K-AKT pathway [47]. Moreover, we also found icariside II induced apoptosis through inhibition of EGF-induced activation of STAT3 in A431 cells [56]. In U937 and U266 cells, Icariside II decreased the phosphorylation of JAK2 and c-Src, the upstream activators of the STAT pathway, and increased the expression of PTPs such as PTEN and protein tyrosine phosphatase (PTP) SH2 domain-containing phosphatase (SHP)-1 [57,59]. Sodium pervanadate (a PTP inhibitor) prevented Icariside II-induced apoptosis as well as STAT3 inactivation in U937 cells. Furthermore, silencing SHP-1 using specific siRNA significantly blocked STAT3 inactivation and apoptosis induced by Icariside II in U937 cells. All these results support a key role of SHP-1 in the suppression of STAT signaling pathways. In addition, icariside II was proved to reduce the level of STAT3 in MDA-MB-231 (breast adenocarcinoma) and DU145 (prostate carcinoma) cells, in which STAT3 is constitutively active [57].

The mitogen-activated protein kinase (MAPK-ERK, also known as Raf-MEK-ERK) pathway consisted several signal factors and the extracellular signal-regulated kinase-1 and 2 (ERK1/2) are extremely important in human cancers, which then activate ERK [95]. The importance of MAPK-ERK pathways in cancer progression and proliferation has been supported by Shield et al. [96]. Icariside II has been shown to inhibit the activation of ERK in melanoma A375 cells [47,48] and epidermoid carcinoma A431 cells [56]. Our data showed that icariside II treatment could effectively inhibit paclitaxel-induced activation of TLR4-MyD88-ERK signaling pathway in human melanoma A375 cells, which is proposed to be a novel target for reversing chemoresistance to paclitaxel. What’s more, icariside II decreased cell proliferation and inactivated Raf-MEK-ERK signaling in osteosarcoma MG-63 and Saos-2 cells [54].

Phosphatidylinostinositol-3-kinase/protein kinase B (PI3K-AKT) signaling was found to play a key role in cell proliferation and is overexpressed in multiple human cancers [97]. As the downstream of PI3K-AKT, the mammalian target of rapamycin (mTOR), is a master growth regulator that senses the presence of growth factors by regulating p70S6K and 4E-binding protein 1 (4E-BP1) [98]. Icariside II, a natural mTOR inhibitor, was found to decrease the phosphorylation of PI3K-PDK1 and dephosphorylated Akt at Thr308 and Ser473 in osteosarcoma MG-63 and Saos-2 cells [54]. Akt activates mTOR by relieving proline-rich Akt substrate 40 (PRAS40)-mediated inhibition of mTOR. In addition, icariside II activated GSK3β, the direct target of Akt, by dephosphorylation atSer9, weakening the stability of transcription factors and cycle-related proteins. The suppression of icariside II on the phosphorylation of PI3K, Akt, PRAS40 was approved by mice bearing osteosarcoma sarcoma-180 cells. In conclusion, icariside II treatment moderated EGF-induced activation of PI3K/Akt/PRAS40 pathway in vitro as well as in vivo [54]. Icariside II was also shown to suppress aberrant energy homeostasis in osteosarcoma U2OS, fibrosarcoma S180 and chondrosarcoma SW1535 cells via suppression of mTORC1 by regulating mTORC1-4E-BP1 axis, suggesting a potential application of icariside II in sarcoma therapy [52]. In addition, icariin was reported to enhance cytotoxicity of doxorubin in the human osteosarcoma doxorubicin (DOX)-resistant MG-63/DOX cell line through down-regulation of PI3K-Akt pathway [65].

### 5.5. Anti-Inflammatory Activity

The hypothesis that chronic inflammation promoted cancer development and progression is strongly supported by the findings that individuals with chronic inflammation of the specific organ are significantly more susceptible to some organ-specific cancers [99]. The induction of myeloid suppressor cells (MDSC), one of the major factors mediating tumor-associated immune suppression, undermines the immune surveillance, thereby providing an environment favorable for tumor growth and allowing proliferation of malignant cells [100]. If inflammation facilitates tumor progression through the induction of more suppressive MDSCby signaling through the toll-like receptor 4 (TLR4) pathway, then it is possible that a decreased pro-inflammatory microenvironment may reduce the potency of MDSC. Data from our laboratory showed that icariin treatment reduced the expression of MRP8/MRP14 and TLR4 on human PBMCs [60]. Administration of icariin inhibited the tumor growth in 4T1-Neu tumor-bearing mice by reducing splenic MDSC accumulation and activation restoration of the functionality of effector CD8+ Tcells [60]. Furthermore, icariin significantly decreased the amounts of nitric oxide and reactive oxygen species in MDSC in vivo. Further, we saw a restoration of IFN-γ production by CD8+T cells and the drops of nitric oxide and reactive oxygen species in tumor bearing mice.

Recent findings demonstrated that cyclooxygenzase-2 (COX-2) is over-expressed in various cancers, including pancreatic cancer [101], gastric carcinoma [102], and prostate cancer [103]. Prostaglandin E_2_ (PGE_2_), the proflammatory product of elevated COX-2, has been shown to play a crucial role in the progression of malignant tumor. Since COX-2 is increased in inflammatory microenvironment, it is considered as a molecular target for cancer prevention and treatment.

The mechanism of icariside II induced apoptosis in hormone-independent prostate carcinoma PC-3 cells was studied in association with COX-2 [40]. Data showed that Icariside II exerted cytotoxicity with IC_50_ of approximately 20 μM on PC-3 cells. Furthermore, icariside II induced apoptosis via the suppression of COX-2, inducible NO synthase (iNOS), vascular endothelial growth factor (VEGF) and mitochondrial membrane potential, release of cytochrome c, and activation of caspase-8, -9 and, -3 expressions, and cleaved PARP. Moreover, exogeneous PGE2 inhibited PARP cleavage and knockdown of COX-2 enhanced PARP cleavage. These results indicated that icariside II induced mitochondrial dependent apoptosis by initiating the inhibition of COX-2/PGE2 pathway in PC-3 prostate [40]. The apoptosis induced by icariside II in acute myeloid leukemia U937 and multiple myeloma U266 was also found to be associated with the decrease of COX-2 [57,59].

## 6. Conclusions

A renewed interest emerges in the study of alternative and less toxic remedies for the treatment of many diseases, including cancer. Maximizing efficacy and minimizing side-effects has been recognized as the major goal of the treatment of cancers. *Herba* *Epimedii* has been traditionally used in clinical due to its multi-purpose activity and low toxicity. Collective data indicate that icariin and icariside II are the main bioactive components of *Herba* *Epimedii* and are potential anticancer agents towards a broad spectrum of human cancers. They exhibit broad toxicity to various types of human cancer cells by interfering with multiple mechanisms, inhibiting multiple signaling pathways, as well as regulating inflammatory microenvironment. Moreover, icariin and icariside II could be used as chemotherapeutic adjuvant agents in combination with standard drugs to improve the treatment effects and avoid drug resistance. In view of these demonstrated effects, icariin and icariside II could be potential therapeutic intervention agents alone or in combination with current chemotherapeutic drugs for cancers.

## Figures and Tables

**Table 1:** Effects and molecular targets of icariin and icariside II on different cancer cell lines.

Cancer Types	Components	Cell Lines	Concentrations		Effects and Molecular Targets	Reference
			Con. Range	IC_50_		
Hepatocellular carcinoma	Icariin	HepG2	10 μM	NA	G0/G1↑, S↓, Bcl-2↓	[37]
		SMMC-7721	5–20 μM	around 10 μM	cleaved caspase-3/9↑, mitochondria cytochrome c↓, cytosol cytochrome c↑, cleaved PARP1↑, XIAP↓, MMP↓, Bcl-2↓, Bax↑, p-JNK↑, ROS↑	[38]
Prostate carcinoma	Icariin	PC-3	30 μM	NA	Cyclin D1↓, CDK4↓	[39]
	Icariside II	PC-3	0–40 μM	around 20 μM	MMP↓, cleaved caspase-3/8/9↑, cleaved PARP↑, COX-2↓, iNOS↓, VEGF↓, PGE_2_↓	[40]
Esophageal cancer	Icariin	EC109	20–80 μM	106.13 μM (12 h)	cleaved caspase-9↑, ROS↑, NADPH oxidase activity↑, GSH↓, GRP78↑, ATF4↑, CHOP↑, p-PERK↑, p-eIF2α↑, Bcl2↓, PUMA↑	[41]
				73.65 μM (24 h)		
				38.59 μM (36 h)		
		TE1	20–80 μM	115.29 μM (12 h)		
				76.77 μM (24 h)		
				42.21 μM (36 h)		
Ovarian cancer	Icariin	A2780	13–50 μM	NA	caspase-3 activity↑, miR-21↓ PTEN↑ RECK↑ Bcl-2↓	[42]
Lung cancer	Icariin	A549	25–100 μM	118.25 μM (12 h)	ROS↑, caspase 3 activity↑, GSH↓, ERS-related molecules↑(p-PERK, ATF6, GRP78, p-eIF2a, and CHOP), Bcl-2↓, PUMA↑	[43]
				86.21 μM (24 h)		
				56.8 μM (36 h)		
	Icariside II	A549	0–20 µM	NA	vimentin↓, N-cadherin↓, NF-κB↓, p-IκBα↓, p65/IκB↑, p-Akt↓ p-GSK-3β↓	[44]
		H1299	0–20 µM	NA		
Melanoma	Icariin	B16	20–200 μg/mL	84.3μg/mL (72 h)	procaspase-9↓ cleaved caspase-9↑	[45]
	Icariside II	A375	0–100 μM	10.6 μM	G0/G1 phase↑, S↓, G2/M arrest↑, cyclin E↓, CDK2↓, cyclin B1↓, P‑CDK1↓, ROS↑, p-p38↑, p-p53↑, p21↑, cleaved caspase-3↑, survivin↓, p-STAT3↓, p-ERK↓, cleaved PARP↑	[46,47,48]
		SK-MEL-5	0–100 μM	11.1 μM		
Leydig cell tumor	Icariin	MLTC-1	12.5–100 μg/mL	50 μg/mL (48 h)	S↓, Bcl-2↓, Bax↑, cytochrome c↑, cleaved caspase-3/9↑, piwil4↓	[49]
Gastric adenocarcinoma	Icariin	BGC-823	20–200 μg/mL	128 μg/mL	Rac1↓, VASP↓	[50]
Medulloblastoma	Icariin	Daoy	NA	NA	Cyclin A↓, CDK2↓, Cyclin B1↓, cleaved caspase-3↑, cleaved caspase-9↑, PARP↑, Bcl-2↓	[51]
		D341	NA	NA		
Sarcoma	Icariside II	U2OS	0–30 µM	NA	4E-BP1↑, mTORC1↓, p-S6K(Thr389)↓, p-S6(Ser235/236)↓, p-4E-BP1 (Ser65)↓	[52]
		SW1353	0–20 µM	NA		
		S180	0–20 µM	NA		
Hepatoblastoma	Icariside II	HepG2	0–30 μM	NA	△ψ_m_↓, ROS↑, Bax/Bcl-2↑, cleaved-Bid↑, LAMP1↑, LMP↑, cleaved caspase-8/9/7/3/PARP↑, LC3B-II↑, SQSTM1↑	[53]
Osteosarcoma	Icariside II	MG-63	10–35 μM	NA	p-EGFR↓, p-PI3K↓, p-Akt↓, p-PDK1↓, p-Raf↓, p-mTOR↓, p-PDK1↓, p-PRAS40↓, p-GSK3β↓, p-ERK↓	[54]
		Saos-2	10–35 μM	NA		
		HOS	0–10 μM	NA	HIF-1α↓, VEGF↓, uPAR↓, ADM↓, MMP2↓, Glut4↓, MCT4↓, aldolase A↓, enolase 1↓	[55]
Epidermoid carcinoma	Icariside II	A431 cell line	0–100 μM	NA	cleaved caspase 9↑, cleaved PARP↑, caspase 9↓, PARP↓, p-STAT3↓, p-ERK↓, p-AKT↑, p-EGFR↑↓	[56]
Acute myeloid leukemia	Icariside II	U937	0–50 μM	NA	cleaved PARP↑, procaspase 3↓, Bcl-2↓, Bcl-X_L_↓, survivin↓, COX-2↓, p-STAT3↓, p-JAK2↓, p-Src↓	[57]
Breast cancer	Icariside II	MCF-7	0–100 μM	72.73 μM (24 h)	MMP↓, cleaved caspase-3/7/8/9↑, cleaved PARP↑, △ψ_m_↓, cytosol cyto c↑, cytosol AIF↑, mitochondrial cyto c↓, mitochondrial AIF↓, Fas↑, FADD↑, Bcl-x_L_↑, Bax↑, BimL↑	[58]
				57.98 μM (48 h)		
				50.95 μM (72 h)		
				37.75 μM (96 h)		
		MDA-MB-231	0–100 μM	97.14 μM (24 h)		
				62.75 μM (48 h)		
				42.40 μM (72 h)		
				38.65 μM (96 h)		
Multiple myeloma	Icariside II	U266	0-100 μM	NA	p-STAT3↓, p-JAK2↓, p-c-Src↓, SHP-1↑, PTEN↑, cyclin D1↓, Bcl-2↓, Bcl-x_L_↓, survivin↓ VEGF↓, COX-2↓, cleaved caspase-3↑, p-PARP↑	[59]

NA, not applicable; ↑, up-regulation; ↓, down-regulation.

**Table 2:** In vivo evaluation of icariin and icariside II in mouse tumor models.

Components	Tumor Models	Transplantation	Treatment	Results	Reference
Icariin	Esophageal cancer EC109	Subcutaneous injection	Given by i.p. 60 and 120 mg/kg every day for 20 days	Significantly inhibit tumor growth	[41]
Icariin	Lung adenocarcinoma A549	Subcutaneous injection	Given by i.p. 100 or 150 mg/kg (5 days/week) for 4 weeks	Significantly inhibit tumor growth	[43]
Icariin	Melanoma B16	Subcutaneous injection into the right flank	Given by p.o. 65 mg/kg every day for 20 days	Apparently inhibit tumor growth	[45]
Icariin	Mammary carcinoma 4 T1-Neu	Subcutaneous inoculation tumor bearing mice	Given by i.p. 100 mg/kg three times a week starting on day 7 until day 28	61% reduction of tumor growth	[60]
Icariin	Hepatoma SMMC-7721	Subcutaneous injectioninto the armpit	Given by i.p. 15, 30, and 60 mg/kg every day for 20 days	38.7%, 54.7%, and 69.9% inhibition in tumor volume, respectively	[38]
Icariin	Hepatoma HepG2	Subcutaneous injection	Given by i.g. 80 mg/kg for 35 days	55.6% inhibition in tumor weight; 47.2% inhibition in tumor volume	[61]
Icariside II	Sarcoma S180	Subcutaneous injection into the right armpit	Given by i.v. 10, 20, 30 mg/kg everyday for 9 days	33.0%, 51.3%, and 62.6% reduction in tumor weight, respectively	[52]
Icariside II	Lung cancer A549	Subcutaneous injection into the flank area	Given by i.v. 30 and 60 mg/kg once every 3 days for 24 consecutive days	Strongly suppress tumor volume	[44]
Icariside II	Liver carcinoma H22	Inoculation	Given by i.v.10, 20, 30 mg/kg everyday for 9 days	Inhibit tumor growth	[53]
Icariside II	Sarcoma S180	Subcutaneous injection into the right flanks	Given by i.p. 10, 20 and 30 mg/kg everyday for 10 days	Inhibit tumor proliferation	[54]
Icariside II	Melanoma B16	Subcutaneous injection into the right flank	Given by i.p. 50 mg/kg and 100 mg/kg 3 times for a week	41% and 49% decrease in tumor volume	[47]

**Table 3:** Icariin and icariside II used as adjuvant agents in combination with hemotherapeutic drugs.

Component	Chemotherapeutic Drugs	Cancer Types	Cell Lines	Tumor Models	Molecular Targets	Reference
Icariin	Temozolomide	Glioblastomamultiforme	U87MG		NF-κB↓	[62]
Icariin	Arsenic Trioxide	Acute promyelocytic leukemia	HL-60	Xenograft murine model (HepG2)	ROS↑	[63,64]
		Hepatocellular carcinoma	NB4			
			SMMC-7721		ROS↑ NF-κB↓cyclin D1↓ Bcl-2↓Bcl-xL↓ COX-2↓survivin↓ VEGF↓	
			HepG2			
Icariin	Doxorubicin	Osteosarcoma	MG-63/DOX		MDR1↓ PI3K/Akt pathway↓	[65]
Icariin	5-Fluorouracil	Colorectal cancer	HT29	Xenograft murine model (HCT116)	NF-κB↓ cyclin D1↓ caspase-8↑ caspase-9↑ caspase-3↑Bax↑ PARP↑Bcl-xL↑	[66]
			HCT116			
Icariin	Gemcitabine	Gallbladder cancer	GBC-SD	Xenograft murine model (GBC-SD)	NF-κB↓ caspase-3↑ G0/G1 phase arrest↑ Bcl-2↓Bcl-xL↓	[67]
			SGC-996			
Icariside II	Paclitaxel	Melanoma	A375		TLR4–MyD88–ERK↓ caspase-3↑ IL-8 ↓ VEGF↓	[48]
Icariside II	Bortezomib	Multiple myeloma	U266		STAT3↓ JAK2↓ c-Src↓ SHP-1↓ PTEN↓ Bcl-2↓Bcl-xL↓survivin↓cyclin D1↓ COX-2↓ VEGF↓	[59]
	Thalidomide		U266			

The empty cells under tumor model indicates the studies are performed on cells (in vitro) rather than on tumor models (in vivo). **↑**, up-regulation; ↓, down-regulation.
